# Indigestible foreign bodies in the forestomach of slaughtered goats in Mogadishu, Somalia

**DOI:** 10.14202/vetworld.2023.1829-1832

**Published:** 2023-09-14

**Authors:** Mohamed A. Shair, Ahmed A. Hassan-Kadle, Aamir M. Osman, Kaltumo M. Y. Ahmed, Abdulkarim A. Yusuf, Ivan R. Barros-Filho, Rafael F. C. Vieira

**Affiliations:** 1 Graduate Program on Veterinary Sciences, Department of Veterinary Medicine, Universidade Federal do Paraná, Curitiba, Paraná, Brazil; 2 Abrar Research and Training Center, Abrar University, Mogadishu, Somalia; 3 Somali One Health Center, Abrar University, Mogadishu, Somalia; 4 Department of Animal Health and Veterinary Services, Ministry of Livestock, Forestry, and Range, Mogadishu, Somalia; 5 Department of Slaughterhouses, Somali Meat Company, Mogadishu, Somalia; 6 Department of Public Health Sciences, University of North Carolina at Charlotte, Charlotte, USA; 7 Center for Computational Intelligence to Predict Health and Environmental Risks (CIPHER), University of North Carolina at Charlotte, Charlotte, USA

**Keywords:** *Capra hircus*, plastic bags, small ruminants, Somali slaughterhouse, Sub-Saharan Africa

## Abstract

**Background and Aim:**

The primary domestic animal in Somali communities is the goat. Their main economic importance is as a food source and a main form of agriculture in the country. There has been a recent decline in the goat population in Somalia, which may be due to the shortage of feed and an increasingly contaminated environment that is affecting the population’s food supply and nutritional status. This study aimed to estimate the prevalence and the factors associated with indigestible foreign bodies (IFBs) ingestion in goats in Mogadishu, Somalia.

**Materials and Methods:**

A cross-sectional study was conducted at the Somalia Meat Company in Mogadishu, Somalia, in February 2022. A total of 250 goats were included in this study, and records were kept on age, sex, body condition, and location. Following the slaughter, goats were inspected for IFBs, and their stomach compartments were incised and examined. Indigestible foreign body classifications was noted and subjected to analysis using the Statistical Package for the Social Sciences version 26.0.

**Results:**

A total of 90/250 (36%; 95% confidence interval [CI]: 30.1–42.3) goats presented IFBs, being 71/90 (79%; 95% CI: 69–87) in the rumen, 12/90 (13%; 95% CI: 7–22) in the reticulum, and seven/90 (8%; 95% CI: 3–15) on both. The most observed IFBs were plastic in 71/90 (79%; 95% CI: 69–87), followed by ropes in eight/90 (10%; 95% CI: 5–18). A high IFB prevalence was observed in goats aged >2–≤3 years (44%), followed by >3 years (36%). The lowest frequency was observed in goats aged <2 years (30%). Overall, there was an association between IFBs in goats and poor body conditions (χ^2^ = 47%, p < 0.04).

**Conclusion:**

The absence of a plastic waste disposal system in the area, and communal free-grazing of livestock in highly contaminated sites, appeared to be significant contributors to the high occurrence of IFBs in goats. Therefore, appropriate policies for solid waste management should be implemented.

## Introduction

Agriculture and livestock farming is the backbone of the Somali economy, contributing over 90% of the country’s total exports and employing over 80% of its population [1]. However, the leading productivity limitations include unpredictable and extreme weather patterns, underdeveloped and fragmented markets, poor value addition, and lack of access to quality inputs such as seeds, fertilizers, and animal vaccines [2]. Goat production in Somalia plays an essential role in national food security and supply. In addition, goat production contributes to sustainable agriculture due to its rapid economic return. It serves as a source of cash income and a living savings account during crop failure and financial distress [3]. Nonetheless, the livestock industry is constrained by various factors such as inadequate technical support services, infrastructure, a marketing system, diseases, and low genetic potential [4].

Ingestion of indigestible foreign bodies (IFBs) in ruminants is a condition that leads to severe economic losses due to high rates of illness and mortality [5]. Ingestion of IFBs may mostly occur during drought, characterized by periods of feed scarcity [6]. In addition, environmental pollution by solid wastes from domestic and commercial sources is prevalent in the least developed countries, including Somalia. Plastic bags, sack threads, ropes, leather, rubber, hair, and plant fibers (bezoars) are the most common IFBs associated with numerous health and economic effects in the livestock industry [4, 6]. The direct economic impact on production results from IFBs may go and lodge in the forestomach of the animals. It may cause rumenitis, rumen impaction, traumatic pericarditis, or traumatic reticuloperitonitis, which may lead to death and indirectly reduce the production of animals [6–8].

A previous study in northern Somalia reported a 33% prevalence of IFBs in goats, with plastic bags, clothes, and robes being the most common [9]. However, there is no available information regarding the occurrence of IFBs in goats from the southern region, the country’s most populous region. Therefore, this study aimed (i) to estimate the prevalence of IFBs in the rumen and reticulum, (ii) to identify the type IFBs, and (iii) to determine the factors associated with IFBs in goats slaughtered in Mogadishu, Somalia.

## Materials and Methods

### Ethical approval and Informed consent

This study was approved by the ethical committee of Abrar University, Somalia (reference number AUEC10521). A written consent was obtained from all the participants.

### Study period and location

**Figure-1:**
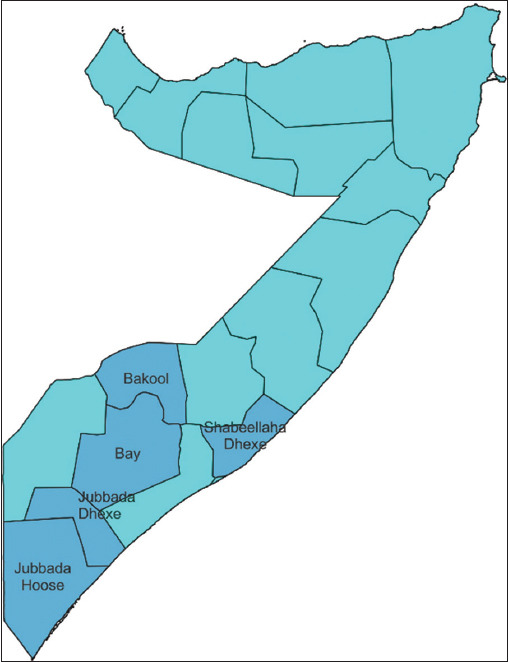
Map of Somalia showing the location of slaughtered goats’ origins. The highlight indicates the locations of the sampled regions [Source: The figure was generated and modified using QGIS software version 3.26.0].

A cross-sectional study was conducted in February 2022 at the Somali Meat Company (SOMEAT) slaughterhouse in Mogadishu City, Somalia. The SOMEAT only slaughters male goats. Goats originated from different regions of the country, mainly the Middle Shabelle, Lower Jubba, Bay, and Bakol regions (Figure-1). The goats were maintained under an extensive free-grazing management system.

### Sample size

A total of 250 goats were included in this study. A structured questionnaire was administered to capture data on age, sex, body condition, and location. The body condition was recorded as poor, moderate, and good based on the goat’s appearance, as described by Steele [10]. Animals were categorized as <2 years, >2–≤3 years, or >3 years.

### Postmortem inspection for IFBs

After the goat was slaughtered, the rumen, omasum, abomasum, and reticulum were examined for IFBs through palpation. Each stomach part was incised and examined, and when IFBs were encountered, they were removed, washed, and identified. The type of IFB was recorded for each site.

### Data management and analysis

Collected data were entered into the Microsoft Excel software^®^ (Microsoft Corporation, Washington, USA) and later transferred and analyzed with Statistical package for the social sciences^®^ statistics software version 26.0 (IBM Corp, Armonk, NY, USA). The Chi-square test was used to evaluate if there is an association between the prevalence of IFBs in goats of different ages, body conditions, and locations. Odds ratio (OR), 95% confidence intervals (95% CI), and p-values were calculated separately for each variable, and results were considered significant when p ≤ 0.05.

## Results

A total of 90/250 (36%, 95% CI = 30–42) goats had IFBs in their forestomach, with eight/90 (9%, 95% CI = 4–17) of them having more than one IFB. The most commonly observed IFB was plastic in 71/90 (79%, 95% CI = 69–87) goats, followed by ropes eight/90 (10%, 95% CI = 5–18), wood five/90 (4%, 95% CI = 1–10), clothing materials five/90 (4%, 95% CI = 1–10), fruit seeds four/90 (4%, 95% CI = 1–10), bones four/90 (4%, 95% CI = 1–10), and leather materials three/90 (3%, 95% CI = 0.6–9) (Table-1).

**Table-1:** Proportion of foreign body types in the rumen and reticulum of goats slaughtered at SOMEAT Slaughterhouse (n = 90).

Organ	Type of foreign body	n	%
Rumen only	Total no. positive	71	---
	Plastic	51	71.8
	Rope	8	11.3
	Cloth	5	7.0
	Wood	5	7.0
	Fruit	4	5.6
	Leather	4	5.6
Reticulum only	Total no. positive	12	----
	Plastic	12	100
Rumen and reticulum	Total no. positive	7	----
	Plastic	7	100

A total of 71/90 (78.9%), 12/90 (13.3%), and seven/90 (7.7%) goats presented IFBs in the rumen, reticulum, and their proportions, respectively (Table-1). None of the IFBs were seen in the omasum and abomasum.

Goats aged >3 years were more likely to have IFBs than <2 years (OR: 1; χ2 = 0.9, p = 0.13) and >2–≤3 years (OR: 2; χ^2^ = 2.7, p = 0.15). The overall IFB prevalence in the poor, moderate, and good body condition group of goats was 27/57 (47.3%, 95% CI = 34–61), 39/111 (35%, 95% CI = 26–45), and 24/82 (29%, 95% CI = 20–40), respectively. Goats with poor body condition were more likely to have IFBs than good (OR: 0.5; χ^2^ = 4.7, p = 0.04) and moderate body condition (OR: 0.6; χ^2^ = 2.4, p = 0.17) (Table-2). When evaluating the goat origin, the highest frequencies of IFBs in the rumen and reticulum were observed in goats from Bakol 14/27 (51.9%, 95% CI = 31.9–71.3). At the same time, the lowest prevalence was found in goats from the Lower Jubba region (Table-1). An association between IFBs occurrence and the goat’s origin was not found (p = 0.27) (Table-2).

**Table-2:** Occurrence of indigestible foreign bodies in different categories of goats slaughtered at SOMEAT Slaughterhouse (n = 250).

Risk factors	Number of slaughtered goats	Positive goats	95% CI	p-value	OR 95%CI
Age					
>3 years	121	44	36.4 (27.8–45.6)	0.43	1.3 (0.7–2.5)
2–3 years	55	24	43.6 (30.3–57.7)	0.15	1.8 (0.9–3.8)
<2 years	74	22	29.7 (19.7–41.5)		
Body condition					
Good	82	24	29.2 (19.7–40.3)	0.04	0.5 (0.2–0.9)
Moderate	111	39	35.1 (26.3–44.8)	0.17	0.6 (0.3–1.1)
Poor	57	27	47.3 (33.9–61)		
Location					
Middle shabelle	28	11	39.3 (21.5–59.4)	0.51	0.6 (0.2–1.8)
Bay	158	54	34.1 (26.8–42.1)	0.13	0.5 (0.2–1.1)
Lower Jubba	37	11	29.7 (15.9–46.9)	0.13	0.4 (0.1–1.1)
Bakol	27	14	51.9 (31.9–71.3)		

CI=Confidence interval, OR=Odds ratio

## Discussion

Ingestion of IFBs in ruminants is a condition that leads to severe economic losses due to the high rates of illness and mortality [5]. In Somalia, limited information about IFBs in ruminants is available. However, such information has never been published in the southern part of the country, which has the largest livestock population. This study revealed that 90/250 (36%, 95% CI = 30–42) goats had IFBs in their forestomach. Therefore, this IFB occurrence level could significantly impact the economy, cause productivity to decline, and potentially increase the risk of animal fatality. The occurrence rate of IFBs in goats recorded during this study is higher than previously reported studies in Kenya [11] and Tanzania [12], with 12% and 18.5% prevalence rates, respectively. However, it was lower than that reported in Ethiopia [13] and Nigeria [14], with 59% and 70% prevalence rates, respectively. The difference in the IFBs prevalence in the studied areas may be attributed to different levels of environmental pollution with IFBs and seasonal variation. A higher IFB prevalence has been associated with high levels of environmental contamination with IFBs, especially in urban areas and in the dry season, where there is a feed shortage, which forces animals to consume anything in their surrounding environment, which may include IFBs [15, 16]. Herein, a more significant proportion of IFBs was observed in the rumen than in the reticulum of goats. This fact may be attributed to the larger rumen size than other stomach parts [17].

Goats aged >3 years were more likely to have IFBs than <2 years (OR: 1; χ^2^ = 0.9, p = 0.13) and >2–≤3 years (OR: 2; χ^2^ = 2.7, p = 0.15). This result agrees with a study conducted in Kenya [11] and differs from a survey conducted in Ethiopia [15]. This difference may be attributed to the gradual ingestion of IFBs over prolonged periods as the animal ages.

Herein, goats with poor body condition were more likely to have IFBs than good (OR: 0.5; χ^2^ = 4.7, p = 0.04) and moderate body condition (OR: 0.6; χ^2^ = 2.4, p = 0.17), in agreement with the previous studies [6, 13]. In contrast, a previous study has reported high prevalence rates in animals in good body condition [11]. This difference may be because once the animal ingests IFBs, the digestive tract may obstruct the passage of food and the absorption of volatile fatty acids, thereby preventing weight gain.

## Conclusion

The ingestion of IFBs is common in goats slaughtered at SOMEAT slaughterhouse in Somalia. Most of the IFBs found were plastic bags. This fact is related to the widespread use of plastic bags in the surrounding environment and inappropriate disposal practices. Our data indicate the need to deploy suitable control measures to reduce environmental pollution’s impact and keep animals from IFBs.

## Authors’ Contributions

MAS, AAH, IRBF, AMO, AAY, KMYA, and RFCV: Designed the study, collected the data, and conducted the methodology. AMO, IRBF, AAH, and RFCV: Performed the data analysis. AMO and RFCV: Drafted the manuscript. All authors have read, reviewed, and approved the final manuscript.
